# Case report: exome sequencing achieved a definite diagnosis in a Chinese family with muscle atrophy

**DOI:** 10.1186/s12883-021-02093-z

**Published:** 2021-03-02

**Authors:** Hui Jiang, Chunmiao Guo, Jie Xie, Jingxin Pan, Ying Huang, Miaoxin Li, Yibin Guo

**Affiliations:** 1 Zhongshan School of Medicine, Sun Yat-sen University, Guangzhou, 510080 China; 2 Key Laboratory of Tropical Diseases Control (SYSU), Sun Yat-sen University, Guangzhou, 510080 China; 3 Center for Precision Medicine, Sun Yat-sen University, Guangzhou, 510080 China; 4 Department of Neurology, The Second Affiliated Hospital, Fujian University of Medical Science, Quanzhou, 362000 China; 5 Department of Hematology, The Second Affiliated Hospital, Fujian University of Medical Science, Quanzhou, 362000 China; 6 The Fifth Affiliated Hospital, Sun Yat-sen University, Zhuhai, 519000 Guangdong China; 7 State Key Laboratory for Cognitive and Brain Sciences, The University of Hong Kong, Hong Kong SAR, China; 8 School of Medicine, Sun Yat-sen University, Shenzhen, 518107 Guangdong China

**Keywords:** Exome sequencing, Diagnosis, *GDAP1*, Charcot-Marie-tooth type 4A, Muscle atrophy, Case report

## Abstract

**Background:**

Due to large genetic and phenotypic heterogeneity, the conventional workup for Charcot-Marie-Tooth (CMT) diagnosis is often underpowered, leading to diagnostic delay or even lack of diagnosis. In the present study, we explored how bioinformatics analysis on whole-exome sequencing (WES) data can be used to diagnose patients with CMT disease efficiently.

**Case presentation:**

The proband is a 29-year-old female presented with a severe amyotrophy and distal skeletal deformity that plagued her family for over 20 years since she was 5-year-old. No other aberrant symptoms were detected in her speaking, hearing, vision, and intelligence. Similar symptoms manifested in her younger brother, while her parents and her older brother showed normal. To uncover the genetic causes of this disease, we performed exome sequencing for the proband and her parents. Subsequent bioinformatics analysis on the KGGSeq platform and further Sanger sequencing identified a novel homozygous *GDAP1* nonsense mutation (c.218C > G, p.Ser73*) that responsible for the family. This genetic finding then led to a quick diagnosis of CMT type 4A (CMT4A), confirmed by nerve conduction velocity and electromyography examination of the patients.

**Conclusions:**

The patients with severe muscle atrophy and distal skeletal deformity were caused by a novel homozygous nonsense mutation in *GDAP1* (c.218C > G, p.Ser73*), and were diagnosed as CMT4A finally. This study expanded the mutation spectrum of CMT disease and demonstrated how affordable WES could be effectively employed for the clinical diagnosis of unexplained phenotypes.

**Supplementary Information:**

The online version contains supplementary material available at 10.1186/s12883-021-02093-z.

## Background

Muscle atrophy comprises progressive conditions that cause loss of muscle mass and weakness in hundreds of different clinical entities. The molecular pathophysiology of these disorders is heterogeneous, with mechanisms ranging from defects in peripheral nervous system, central nervous system to the neurotrophic deficiency [1]. Some cancers, chronic inflammatory diseases, and acute critical illness patients are also accompanied by symptoms of muscle atrophy [2]. The highly overlapping muscle atrophy phenotypes among numerous conditions almost inevitably lead to insufficient disease knowledge and diagnostic errors [3]. Besides, many genetic diseases had multiple causal genes, such as limb-girdle muscular dystrophy [4], amyotrophic lateral sclerosis [5] and nemaline myopathy [6]. Moreover, mutations in the same gene can also lead to different forms of phenotype abnormity. For example, mutations in lamin A/C (*LMNA*) gene can result in Emery-Dreifuss muscular dystrophy, dilated cardiomyopathy, Charcot-Marie-Tooth (CMT) disease and spinal muscular atrophy [7]. The complicated genetic and phenotypic heterogeneity poses substantial obstacles to a rapid and accurate diagnosis in the conventional workup, and makes patients embark on a “diagnostic odyssey”.

Next-generation sequencing (NGS) technologies are revolutionizing the field of genetic diagnosis by making massively parallel sequencing much more efficient and affordable [8]. Compare with whole-genome sequencing (WGS), whole-exome sequencing (WES) only analyzes coding regions that compose less than 2% of the human genome but harbour around 80% known Mendelian disease-associated variants [9]. Coupled with computational bioinformatics tools, WES has emerged as a rapid, unbiased and cost-effective sequencing strategy for elucidating genetic variants underlying human diseases [10]. A typical design of WES is to use a family trio that contains a patient and healthy parents. Such design enables rare benign familial variants to be filtered out efficiently, de novo mutations to be easily identified, and candidate variants to be prioritized under established inheritance pattern. The efficient analysis can improve the diagnostic rate for genetically heterogeneous disorders, such as complex neurologic diagnosis and multiple congenital anomalies [11, 12].

In this study, we reported the usage of exome sequencing and comprehensive bioinformatics analyses to elucidate the underlying genetic cause and reach a clinical diagnosis for a suspected familial amyotrophy from China.

## Case presentation

### Clinical features of the studied family

**Fig. 1:**
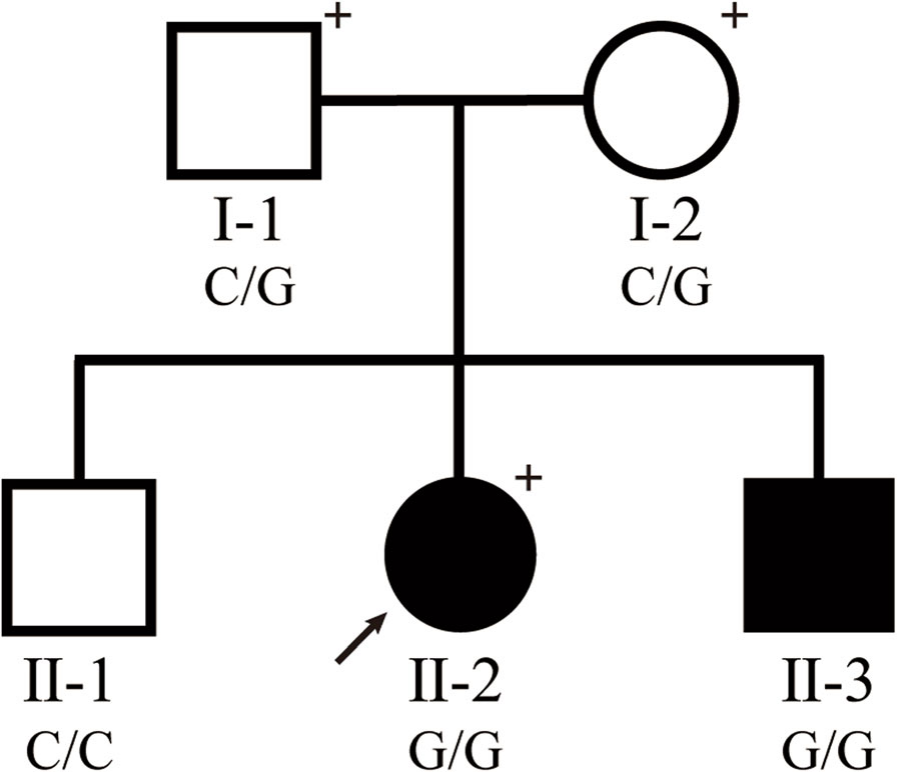
Pedigree diagram of the studied family. Squares and circles represent males and females. Filled and unfilled symbols indicate affected and unaffected individuals. The proband is marked with an arrow, and individuals with whole-exome sequencing are marked with “+” at the upper right corner. The genotypes at the mutation of *GDAP1* c.218C > G, p.Ser73* are listed below each individual

**Fig. 2:**
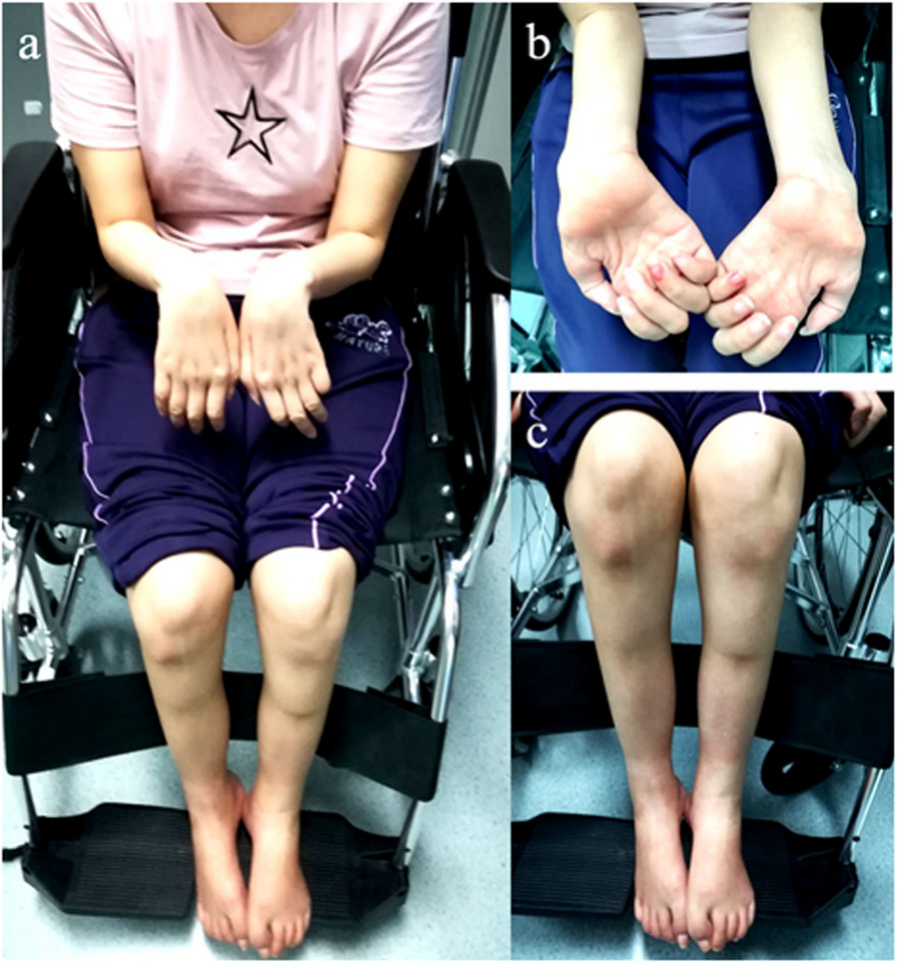
Characteristics of the proband. (**a**) Muscular atrophy and distal skeletal deformity lead the proband to a wheelchair-dependent life; (**b**) Severe weakness and atrophy of lower arm muscles, valgus wrists and contracted palms; (**c**) Weakness and atrophy of lower leg muscles, feet droop

The Chinese family with genetic muscle atrophy was from Guangdong province (Fig. 1 for the pedigree diagram). The proband (II-2) was a 29-year-old female who presented with severe amyotrophy and distal skeletal deformity that plagued her family over 20 years. A stumbling gait was first noticed at the age of 5 and gradually developed with ankle contracture and foot bending. Acupuncture treatment was used, and surgery was carried out at the age of 10, but conditions did not get better. Instead, without getting enough exercise after the surgery, her feet became weak, and extremity muscle wasting got worse, which led her to a wheelchair-dependent life. Physical examination recently revealed her bilateral muscle weakness and atrophy of both her lower and upper limbs, accompanied by distal deformities of severe valgus ankles and wrists, contracted feet and palms (Fig. 2). No other aberrant symptoms were detected in speaking, hearing, vision, and intelligence. Similar symptoms were also found on her younger brother (II-3, Supplementary Fig. S1), while both her parents (I-1 and I-2) and her older brother (II-1) were normal, indicating that genetic factors might play an important role in the emergence of this familial disease. Before they came to our laboratory for counselling, a series of clinical checks and gene panel detections of genetic disorders with similar phenotypes, such as *BMP1* for osteogenesis imperfecta, type XIII (MIM: 614856), *HOXD13* for Brachydactyly-syndactyly syndrome (MIM: 610713), and *SEC24D* for Cole-Carpenter syndrome 2 (MIM: 616294), have been performed to find the causal variants for the disease in this family. However, all the results turned out to be negative. Conventional workup failed to make a definite diagnosis for such a disease with heterogeneous and non-specific clinical features.

### Identification of candidate mutations by trio-exome sequencing

**Fig. 3:**
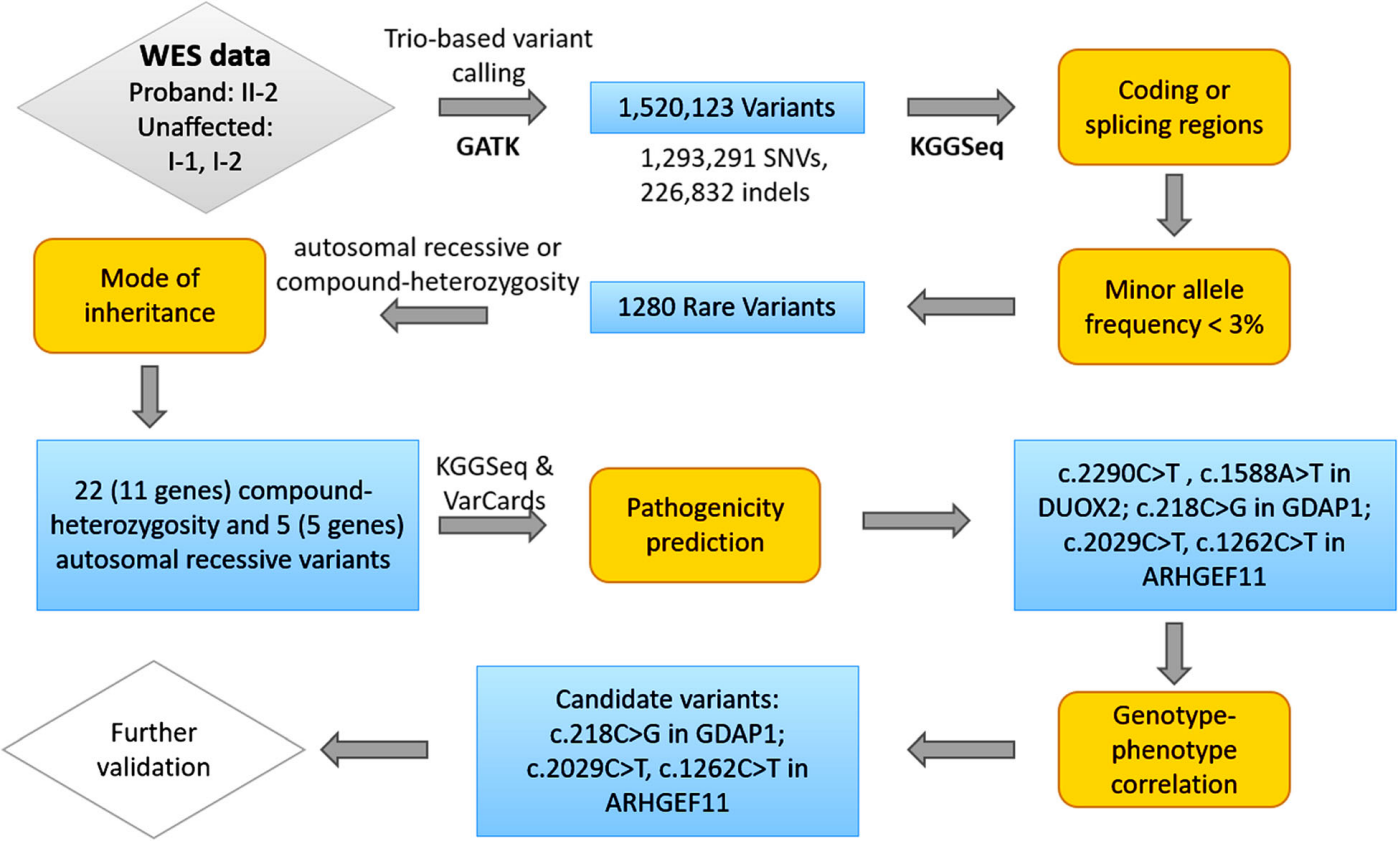
The workflow of discovering disease-causing variants for the studied family

To uncover the genetic variants contributing to this disease, we performed trio-exome sequencing on the proband II-2 and her unaffected parents I-1 and I-2. More than 90% of the targeted regions were sequenced with a coverage ≥30X. An identical by descent (IBD)-based genetic relationship was estimated using the software KING [13], the proportion of shared IBD genome segments between I-1 and I-2 was estimated to be nearly zero, suggesting that the parents were not consanguineous. The steps of genetic variant analysis were illustrated in Fig. 3. Raw read pairs were mapped onto the University of California, Santa Cruz (UCSC) human reference genome (version hg19) by using Burrows-Wheeler Aligner (BWA) with standard parameters [14]. Duplicated reads were marked by Picard (http://broadinstitute.github.io/picard/). Regional realignment and quality score recalibration were carried out by using Genome Analysis Toolkit (GATK) [15] with Best Practices recommended parameters to call single-nucleotide variants (SNVs) and short insertion/deletion variants (Indels). In total, the GATK pipeline called 1,520,123 variants (including 1,293,291 SNVs and 226,832 Indels), at which at least one family member had an allele different from the reference genome.

A software platform KGGSeq (http://pmglab.top/kggseq/) [16, 17] was used to prioritize candidate variants by three levels’ filtration and annotation (genetic level, variant-gene level and knowledge level). After excluding the intronic variants, synonymous variants, variants with minor allele frequency ≥ 3% based on 1000 Genomes Project and gnomAD databases and variants that did not match the suspected inheritance pattern of autosomal recessive or compound-heterozygosity, 27 variants in 16 genes were retained. Details of these variants were summarized in Supplementary Table S1. Then we used KGGSeq and VarCards (http://varcards.biols.ac.cn/) to predict whether these variants were deleterious. Variants with “Y” in “KGGSeq Integrated prediction” or a prediction score higher than 0.7 in VarCards [18] were considered as being deleterious. For compound-heterozygosity inherited variants, two deleterious variants on the same gene were considered for further study. Finally, we manually prioritized variants in the gene associated with known amyotrophy diseases or phenotypes by literature survey. As a result, heterozygous variants c.2029C > T, p.Arg677Cys and c.1262C > T, p.Thr421Met of *ARHGEF11* [19], and homozygous nonsense mutation c.218C > G, p.Ser73* of *GDAP1* [20] were selected for Sanger sequencing validation.

### Validation of the candidate mutations

**Fig. 4:**
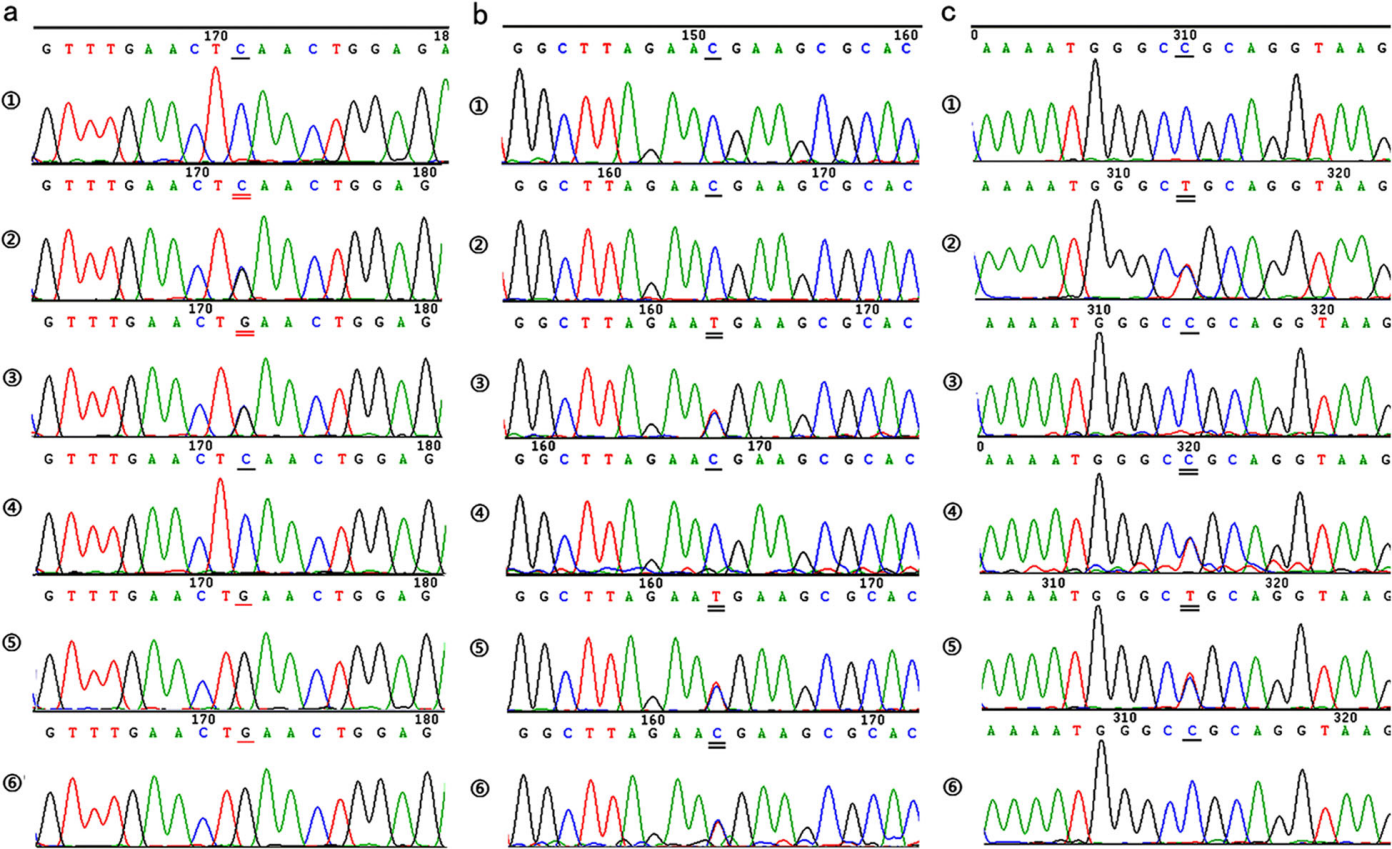
Sanger sequencing and co-segregate validation of candidate mutations. Sanger sequencing results of (**a**) the c.218C > G, p.Ser73* mutation of *GDAP1*; (**b**) the c.1262C > T, p.Thr421Met mutation of *ARHGEF11*; (**c**) the c.2029C > T, p.Arg677Cys mutation of *ARHGEF11*. ①-⑥ are represented as genotypes of normal control, I-1, I-2, II-1, II-2 and II-3, respectively

Candidate variants were validated by testing the genotype’s co-segregation with the phenotype in all family members, using conventional Sanger sequencing. The primers for candidate variants are listed in Supplementary Table S2. As shown in Fig. 4, the nonsense mutation c.218C > G (p.Ser73*) of *GDAP1* was found to co-segregate with the disease phenotype in the family. All the affected individuals had alternative allele homozygous genotype. In contrast, the unaffected individuals had either heterozygous or reference allele homozygous genotype at this variant, concordant with the autosomal recessive inheritance pattern (Fig. 4a). However, heterozygous missense variants of *ARHGEF11* (c.2029C > T, p.Arg677Cys and c.1262C > T, p.Thr421Met) did not co-segregate with disease phenotype under the hypothesis of compound-heterozygosity inheritance model (Fig. 4b, c).

**Fig. 5:**
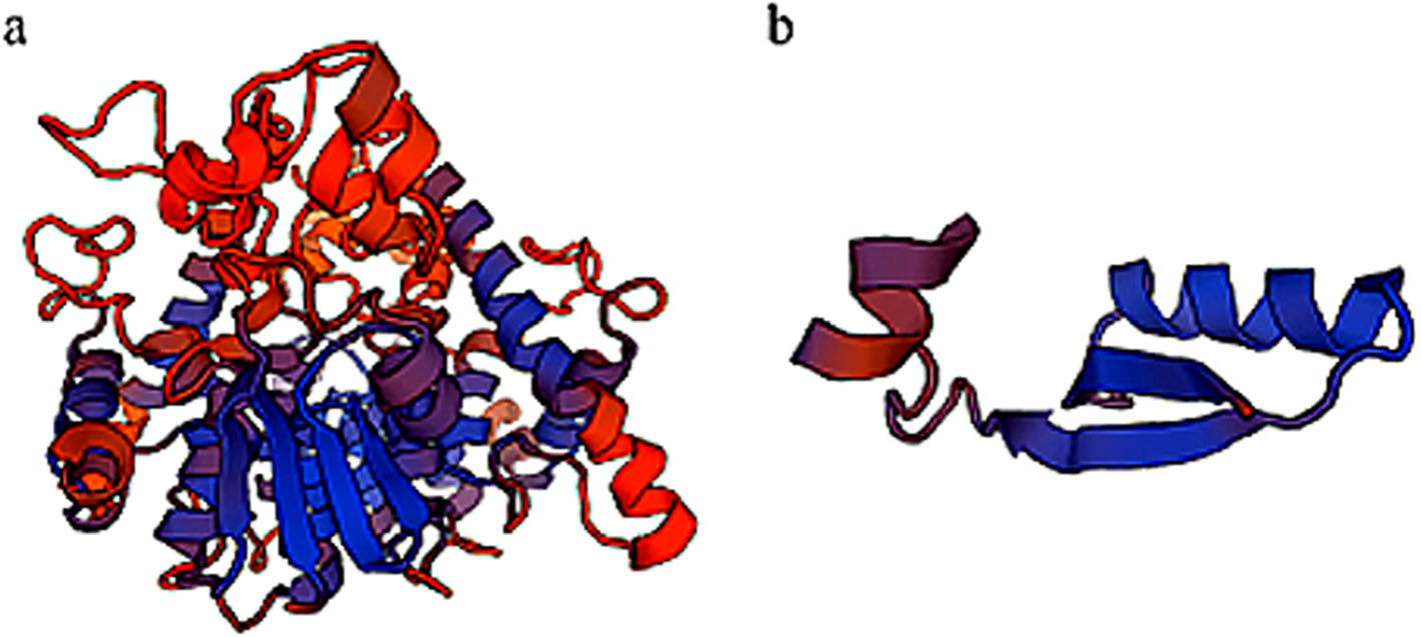
Swiss-model modelling of *GDAP1* mutation. (**a**) Wild type protein structure; (**b**) Predicted p.Ser73* mutant type protein structure

The nonsense mutation in the *GDAP1* gene (Entrez gene ID: 54332), was a C to G transversion at position chr8: 75,263,609 (rs764229116) that leads to a stop-gain codon (p.Ser73*) in the second exon of *GDAP1* gene (NM_018972 and NM_001040875). The Ser73 residue locates in a highly phylogenetically conserved region among mammals (Supplementary Fig. S2). According to the predicted structure by Swiss-model [21] (https://swissmodel.expasy.org/), this nonsense variant might truncate protein product of *GDAP1* from 358 amino acids to 72 amino acids (Fig. 5), which also suggested that the mutation is damaging functionally. Therefore, *GDAP1* (c.218C > G, p.Ser73*) mutation was considered the most likely causal variant for this familial disease.

### Improvement of diagnosis according to the genetic finding

*GDAP1* encodes a member of the ganglioside-induced differentiation-associated protein family, which may play a role in a signal transduction pathway during neuronal development. Mutations in this gene have been associated with various forms of CMT [22–25], a common inherited peripheral neurological disorder characterized by muscle wasting, weakness, and sensory loss (usually most severe distally) [26]. Especially, patients carrying *GDAP1* truncating mutations tend to have a more severe phenotype and generally become wheelchair users before age 40 [27]. The patients of this family also had severe phenotypes. To verify the suggested diagnosis of the nonsense mutation in *GDAP1*, we examined electromyography (EMG) and nerve conduction velocity (NCV) on the two affected individuals of this family. The results were summarized in Supplementary Table S3. No motor or sensory responses could be elicited in the proband II-2. In patient II-3, except for weak motor action potentials in the left femoral and peroneal nerves, no other motor or sensory action potentials were obtained. EMG showed widen motor unit action potential (MUAP) time limit, increased amplitude, large polyphasic potentials and reduced recruitment patterns in multiple muscles, such as deltoid, iliopsoas and T7 paraspinal muscles. These results suggested severe peripheral neurogenic impairments in the upper and lower limbs of the patients. The skeletal X-ray imaging was also conducted on the proband to check for bone abnormalities of the extremities. Except for a slightly reduced bone mineral density, no abnormal bone structure and dysplasia were found in bilateral hands and feet (Supplementary Fig. S3), suggesting that the valgus ankles and wrists may not be caused by bone dysplasia. Based on the clinical features, NCV and EMG results, and mutation screening results, a diagnosis of CMT type 4A was suspected. The homozygous nonsense variation *GDAP1* (c.218C > G, p.Ser73*) in the CMT type 4A patients was not previously reported.

## Discussion and conclusions

The combination of WGS or WES with bioinformatics analysis is a very effective strategy for unravelling Mendelian genetic disease genes. The more cost-effective strategy, WES, often help diagnose genetic diseases with atypical clinical manifestations, which may play an important role in early diagnosis, prenatal and postnatal care, medication guidance and new drug development of hereditary diseases. In this study, we reported two affected siblings who presented with severe amyotrophy and distal skeletal abnormality, but failed to be diagnosed by conventional workup initially. By using a trio WES approach and a comprehensive bioinformatics prioritization framework implemented in KGGSeq for downstream analysis, we detected a nonsense mutation (c.218C > G, p.Ser73*) of Ganglioside-induced differentiation associated protein 1 (*GDAP1*) that may be responsible for muscle atrophy in patients of this family.

*GDAP1* mainly expressed in nervous tissues, encodes an integral, tail-anchored protein of 358 amino acids located at the mitochondrial outer membrane and the peroxisomal membrane [28]. The protein plays a role in several mitochondrial functions, including mitochondrial dynamics, redox processes, mitochondrial transport, calcium homeostasis, and energy production [29, 30]. Mutations in *GDAP1* show a wide range of phenotypic and genetic heterogeneity, leading to subtypes of Charcot–Marie–Tooth (CMT) disease, including autosomal recessive (CMT4A [22] and AR-CMT2K [24]) and autosomal dominant (AD-CMT2K [23]). In the Clinvar database (https://www.ncbi.nlm.nih.gov/clinvar), there have been 153 non-synonymous mutations of *GDAP1* linked with CMT, among which 11 are nonsense mutation. Nonsense mutations of *GDAP1* are usually associated with severe early neuropathy [31], while missense mutations may result in a slightly milder progression and dominant phenotype [27, 32]. Recently, a homozygous *GDAP1* variant (c.667_671dup) leading to a premature termination codon (p.Gln224Hisfs*37) was reported on a severe early-onset CMT in a Vietnamese family under a recessive inheritance model [33]. In our study, the responsible nonsense mutation of *GDAP1* (c.218C > G) leads to a premature stop codon (p.Ser73*) that shortens the protein product of *GDAP1* from 358 amino acids to 72 amino acids. The patients’ clinical features in the studied Chinese family were consistent with the severe peripheral neuropathy of CMT4A. The p.Ser73* variant revealed by the present study has not been described elsewhere and thus updates the mutational spectrum of CMT, especially in the Chinese population.

CMT is a group of inherited peripheral neuropathies affecting motor and sensory neurons, with an estimated prevalence of about 1:3300 [34]. Although mutations in over 90 genes were known to be associated with CMT, the genetic cause of CMT remains unclear in more than 50% of affected individuals [35]. CMT clinical features are diverse, ranging from severe defects in early childhood to only mild features in very late life [36]. Dominant, recessive or dual pathologic alleles on autosomal or X-chromosome were identified in different CMT cases [37, 38]. Even for mutations in the same gene, phenotypes can also be varied. For such a disorder with high genetic and phenotypic heterogeneity, conventional polymerase chain reaction–Sanger sequencing methods are usually inefficient for diagnostic testing [39]. In contrast, whole exome analysis appears to be a rather efficient strategy concerning accuracy, speed, and cost [40]. In the present study, our WES revealed a novel nonsense mutation of *GDAP1* as a causing variation and subsequently facilitated a quick diagnosis of CMT4A for the patients. As *PMP22* and *GJB1* are the top 2 frequent genes associated with CMT [41, 42], we also checked whether there are variations in them. However, neither *PMP22* nor *GJB1* had rare damaging mutations in the proband, which also helped exclude CMT1A and CMTX1. Understanding the molecular basis of CMT is important for effective genetic counselling, management, treatment, and prenatal testing.

In summary, we have successfully applied exome sequencing for a previously unexplained familial disease to a final diagnosis with Charcot-Marie-Tooth type 4A, and identified a novel *GDAP1* mutation (c.218C > G, p.Ser73*) responsible for CMT disease. These findings suggested that WES can be a highly effective diagnostic method for clinically heterogeneous disorders like CMT.

## Supplementary Information

**Additional file 1.**

## Data Availability

The exome sequencing datasets generated during the current study are not publicly available due to patient privacy and legal issues but are available from the corresponding author on reasonable request.

## Abbreviations

WGS: Whole-genome sequencing
WES: Whole-exome sequencing
CMT: Charcot–marie–tooth
CMT4A: Charcot-marie-tooth type 4A
*GDAP1*: Ganglioside-induced differentiation associated protein 1
GATK: Genome analysis toolkit
*ARHGEF11*: Rho guanine nucleotide exchange factor 11
EMG: Electromyography
NCV: Nerve conduction velocity
MUAP: Motor unit action potential
*PMP22*: Peripheral myelin protein 22
*GJB1*: Gap junction protein beta 1.
