# Supplementary material for: Case report: exome sequencing achieved a definite diagnosis in a Chinese family with muscle atrophy
Source: BMC Neurol. 2021 Mar 2;21:96. doi: 10.1186/s12883-021-02093-z (PMC7923504; doi:10.1186/s12883-021-02093-z)
Supplement: Supplementary file 1 — Additional file 1. [file 12883_2021_2093_MOESM1_ESM.docx]

**Supplementary Materials**

**Supplementary Files**

**
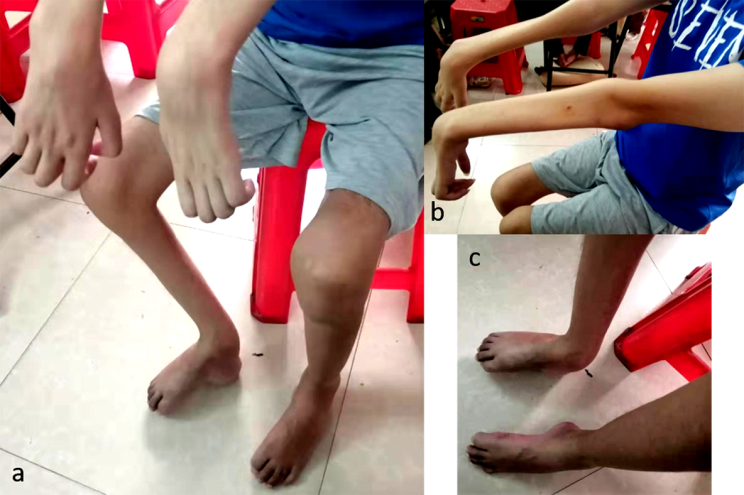
**

**Figure S1**. Characteristics of the proband’s younger brother. (a) Muscular atrophy and distal skeletal deformity; (b) Severe weakness and atrophy of lower arm muscles, valgus wrists and contracted palms; (c) Weakness and atrophy of lower leg muscles, contracted feet.


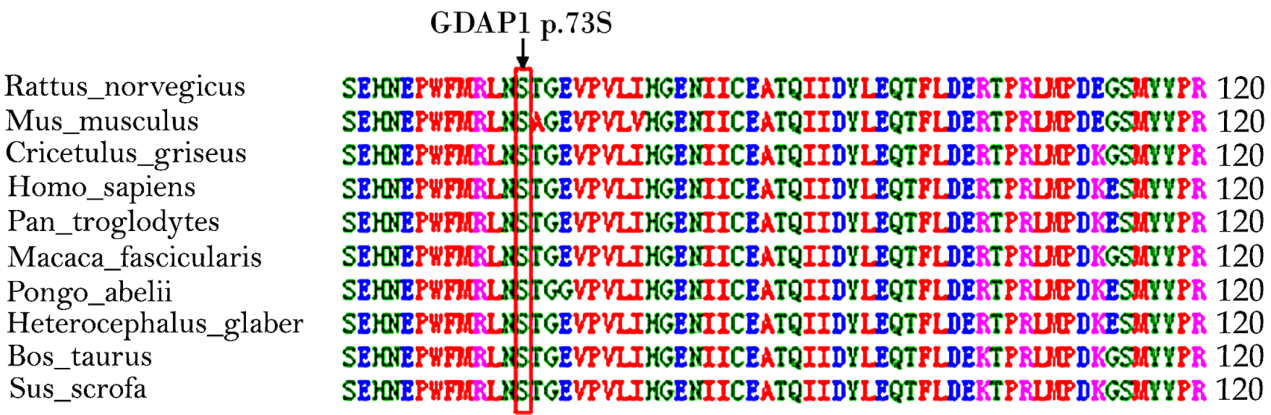


**Figure S2**. Conservation analysis of GDAP1 among different mammalian species performed using CLUSTAL Omega. p.73Ser was highly conserved in 10 mammalian species.


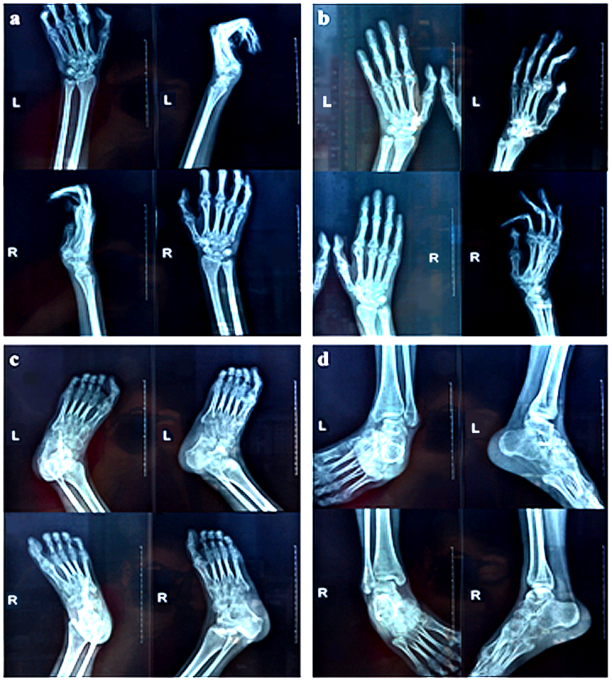


**Figure S3**. Skeletal X-ray images of the proband. (a) Positive side and left-right oblique position of the double wrist joint. No abnormalities. (b) Positive oblique position of the hands. The interphalangeal joint of the thumb and the proximal interphalangeal joint of the index finger are deformed. (c) Positive oblique position of the feet. Osteoporosis. (d) Positive oblique position of the ankle joint. Double ankle valgus deformity, osteoporosis.

**Supplementary Tables**

**Table S1**. Candidate mutations of the muscular atrophy family identified by whole-exome sequencing.

| **Gene** | **Location** | **NM number** | **cDNA** | **Protein** | **rsID** | **VariantType** | **Ⅱ-2 genotype** | **MAF** | **SIFT** | **PolyPhen2** | **KGGSeq integrated score** | **KGGSeq Integrated prediction** | **VarCards damaging score** |
| --- | --- | --- | --- | --- | --- | --- | --- | --- | --- | --- | --- | --- | --- |
| **ARHGEF11** | 1q21 | NM_014784 | c.2029C>T | p.R677C | rs193163419 | missense | Heterozygous | 0.0062 | 0 | 1 | 0.277 | Y | 0.74 |
|  | 1q21 | NM_014784 | c.1262C>T | p.T421M | rs200642355 | missense | Heterozygous | 0.0026 | 0.008 | 1 | 0.0236 | N | 0.78 |
| **XIRP2** | 2q24.3 | NM_152381 | c.419T>C | p.I140T | rs74698684 | missense | Heterozygous | 0.0161 | 1 | 0.001 | 0.0094 | N | 0.15 |
|  | 2q24.3 | NM_152381 | c.4004C>A | p.T1335N | rs184341388 | missense | Heterozygous | 0.0063 | 0.161 | 0.952 | 0.07 | N | 0.7 |
| **FGF2** | 4q28.1 | NM_002006 | c.17G>A | p.G6D | rs201172719 | missense | Heterozygous | 0.024 | 0.015 | 1 | 0.0022 | N | 0.2 |
|  | 4q28.1 | NM_002006 | c.491G>A | p.R164Q | 4:123748421 | missense | Heterozygous | NA | NA | 0.637 | 0.06 | N | 0.55 |
| **PCDHGB7** | 5q31.3 | NM_032101 | c.1510T>G | p.Y504D | rs532216579 | missense | Homozygous | 0.024 | 0.105 | 0.926 | 0.0989 | Y | 0.55 |
| **DNAH11** | 7p15.3 | NM_001277115 | c.2542G>A | p.V848M | rs80328282 | missense | Heterozygous | 0.0219 | NA | 0.996 | 0.0011 | N | 0 |
|  | 7p15.3 | NM_001277115 | c.8275T>C | p.F2759L | rs148656615 | missense | Heterozygous | 0.0026 | 0.073 | 0.533 | 0.0532 | N | 0.65 |
| **SDK1** | 7p22.2 | NM_152744 | c.238C>T | p.R80C | rs369731384 | missense | Heterozygous | 0.0235 | 0.101 | 0.998 | 0.0017 | N | 0.05 |
|  | 7p22.2 | NM_152744 | c.2161G>A | p.V721I | rs138116831 | missense | Heterozygous | 0.0154 | 0.140 | 0 | 0.0019 | N | 0.17 |
| **GDAP1** | 8q21.11 | NM_018972 | c.218C>G | p.S73* | rs764229116 | Stop-gain | Homozygous | 0.00005 | 0.179 | 0.039 | NA | NA | 1 |
| **MPDZ** | 9p23 | NM_001261407 | c.3032T>C | p.I1011T | rs192785156 | missense | Heterozygous | 0.0016 | NA | NA | 0.0522 | N | 0.57 |
|  | 9p23 | NM_001261407 | c.2429G>C | p.C810S | rs200535644 | missense | Heterozygous | 0.0062 | 0.006 | 0.348 | 0.0013 | N | 0.09 |
| **CUBN** | 10p13 | NM_001081 | c.4770C>G | p.I1590M | rs529907907 | missense | Heterozygous | 0.0019 | 0.346 | 0.006 | 0.0214 | N | 0.52 |
|  | 10p13 | NM_001081 | c.2257G>A | p.V753M | rs77886913 | missense | Heterozygous | 0.0074 | 0.029 | 0.975 | 0.0019 | N | 0.04 |
| **WDFY4** | 10q11.23 | NM_020945 | c.4714G>A | p.G1572R | rs190274930 | missense | Heterozygous | 0.0105 | 0.322 | 0.875 | 0.0018 | N | 0.09 |
|  | 10q11.23 | NM_020945 | c.7195G>A | p.V2399M | rs142643091 | missense | Heterozygous | 0.0187 | 0.532 | 0.054 | 0.2466 | Y | 0.68 |
| **LRRK2** | 12q12 | NM_198578 | c.2264C>T | p.P755L | rs34410987 | missense | Heterozygous | 0.0093 | 0.002 | 1 | 0.1379 | Y | 0.48 |
|  | 12q12 | NM_198578 | c.7153G>A | p.G2385R | rs34778348 | missense | Heterozygous | 0.0289 | 0.597 | 0.647 | 0.0083 | N | 0.27 |
| **DUOX2** | 15q21.1 | NM_014080 | c.2290C>T | p.R764W | rs141291775 | missense | Heterozygous | 0.0009 | 0.188 | 0.074 | 0.7839 | N | 0.74 |
|  | 15q21.1 | NM_014080 | c.1588A>T | p.K530* | rs180671269 | Stop-gain | Heterozygous | 0.0095 | NA | 1 | NA | NA | 1 |
| **USP8** | 15q21.2 | NM_005154 | c.199G>A | p.V67M | rs201994026 | missense | Heterozygous | 0.0006 | NA | NA | 0.0057 | N | 0.43 |
|  | 15q21.2 | NM_005154 | c.2302G>C | p.V768L | 15:50784965 | missense | Heterozygous | 0.00005 | 0.124 | 0.723 | 0.0136 | N | 0.48 |
| **TIGD7** | 16p13.3 | NM_033208 | c.968C>G | p.T323S | rs185294194 | missense | Homozygous | 0.0173 | 0.009 | 0.004 | 0.0033 | N | 0.23 |
| **DHRS7B** | 17p11.2 | NM_015510 | c.115C>T | p.R39W | rs200479871 | missense | Homozygous | 0.0034 | 0.126 | 0.006 | 0.0151 | N | 0.3 |

**Table S2**. PCR primer sequences for candidate variants.

| Variants | Forward Primer (5’-3’) | Reverse Primer (5’-3’) | ProductSize (bp) |
| --- | --- | --- | --- |
| *ARHGEF11* c.1262C>T | CACGACTACAGGTTGGC | GGGGAGAGGTTGAAAGA | 265 |
| *ARHGEF11* c.2029C>T | TTCCCAAGTCCCAAAGTC | ACCAAACAGAGGCACCAC | 289 |
| *GDAP1* c.218C>G | GGTGTCCAGGGAAGTCATTT | AAAGTAAATCCAAGAAATCGCAAG | 203 |

**Table S3**. Summary of the electromyography and nerve conduction velocity findings of the affected individuals.

S3-1 Motor nerve conduction

| Individuals | II-2 | | | | II-3 | | | |
| --- | --- | --- | --- | --- | --- | --- | --- | --- |
| Sex/age | Female/29 | | | | Male/21 | | | |
|  | DML  (ms) | CMAP amplitude  (mV) | Ref. DML  (ms) | Ref. CMAP amplitude  (mV) | DML  (ms) | CMAP amplitude  (mV) | Ref. DML  (ms) | Ref. CMAP amplitude  (mV) |
| Ulnar nerve (right) |  | | | | | | | |
| Wrist-ADM | —— | —— | < 3.0 | > 8.0 | —— | —— | < 2.9 | > 8.0 |
| Median nerve (right) |  | | | | | | | |
| Wrist-APB | —— | —— | < 3.8 | > 9.0 | —— | —— | < 3.7 | > 9.0 |
| Femoral nerve (left) |  | | | | | | | |
| Grion-rectus abdominis | —— | —— | > 4.9 | > 5.7 | 4.79 | 3.5 | > 4.9 | > 5.7 |
| Femoral nerve (right) |  | | | | | | | |
| Grion-rectus abdominis | —— | —— | > 4.9 | > 5.7 | —— | —— | > 4.9 | > 5.7 |
| Tibial nerve (left) |  | | | | | | | |
| Ankle-AH | —— | —— | > 2.7 | > 25.0 | —— | —— | > 3.0 | > 32.0 |
| Tibial nerve (right) |  | | | | | | | |
| Ankle-AH | —— | —— | > 2.7 | > 25.0 | —— | —— | > 3.0 | > 32.0 |
| Common peroneal nerve (left) |  | | | | | | | |
| Extensor digitorum brevis | —— | —— | < 3.7 | > 6.0 | —— | —— | < 3.6 | > 6.0 |
| Tibialis anterior | —— | —— | < 3.7 | > 6.0 | 3.7 | 2.9 | < 3.6 | > 6.0 |
| Common peroneal nerve (right) |  | | | | | | | |
| Extensor digitorum brevis | —— | —— | < 3.7 | > 6.0 | —— | —— | < 3.6 | > 6.0 |
| Tibialis anterior | —— | —— | < 3.7 | > 6.0 | —— | —— | < 3.6 | > 6.0 |

DML: distal motor latency; CMAP: compound muscle action potential; Ref. DML: normal reference value of DML; Ref. CMAP amplitude: normal reference value of CMAP amplitude. “——” represent no corresponding signal was detected.

S3-2 Sensory nerve conduction

| Individuals | II-2 | | II-3 | |
| --- | --- | --- | --- | --- |
| Sex/age | Female/29 | | Male/21 | |
|  | SNAP amplitude (uV) | Ref. SNAP amplitude (uV) | SNAP amplitude (uV) | Ref. SNAP amplitude (uV) |
| Ulnar nerve (right) |  | | | |
| finger V-Wrist | —— | > 7.1 | —— | > 7.2 |
| Median nerve (right) |  | | | |
| finger I-Wrist | —— | > 21.0 | —— | > 24.7 |
| finger III-Wrist | —— | > 9.5 | —— | > 11.3 |
| Common peroneal nerve (left) |  | | | |
| ankle-fibulae capitulum | —— | > 0.8 | —— | > 0.9 |
| Common peroneal nerve (right) |  | | | |
| ankle-fibulae capitulum | —— | > 0.8 | —— | > 0.9 |
| Sural nerve (left) |  | | | |
| middle of calf-lateral malleolus | —— | > 3.3 | —— | > 4.3 |
| Sural nerve (right) |  | | | |
| middle of calf-lateral malleolus | —— | > 3.3 | —— | > 4.3 |

SNAP: sensory nerve action potential; Ref. SNAP amplitude: normal reference value of SNAP amplitude. “——” represent no corresponding signal was detected.

S3-3 Electromyography

| Individuals | II-2 | | | | | | II-3 | | | | | |
| --- | --- | --- | --- | --- | --- | --- | --- | --- | --- | --- | --- | --- |
| Sex/age | Female/29 | | | | | | Male/21 | | | | | |
|  | Insertion Activity | Spontaneous Activity | | | | Voluntary Activity | Insertion Activity | Spontaneous Activity | | | | Voluntary Activity |
|  |  | Fib | PSW | Fasc | CRD | IP |  | Fib | PSW | Fasc | CRD | IP |
| Rectus femoris (left) | Normal | 0/10 | 0/10 | 0 | 0 | Unable to shrink | Normal | 0/10 | 0/10 | 0 | 0 | Simple |
| Iliopsoas (right) | Normal | 0/10 | 0/10 | 0 | 0 | Simple | Normal | 0/10 | 0/10 | 0 | 0 | Simple |
| Iliopsoas (left) | Normal | 0/10 | 0/10 | 0 | 0 | Simple-Mixed | NP | NP | NP | NP | NP |  |
| Paraspinal muscle T7(right) | Normal | 0/10 | 0/10 | 0 | 0 |  | Normal | 0/10 | 0/10 | 0 | 0 |  |
| Sternocleidomastoid (left) | Normal | 0/10 | 0/10 | 0 | 0 | Mixed | Normal | 0/10 | 0/10 | 0 | 0 | Mixed |
| Bicipital muscle of arm (left) | Normal | 0/10 | 0/10 | 0 | 0 | Simple-Mixed | NP | NP | NP | NP | NP |  |
| Deltoid (right) | Normal | 0/10 | 0/10 | 0 | 0 | Simple-Mixed | Normal | 0/10 | 0/10 | 0 | 0 | Simple-Mixed |
| Deltoid (left) | Normal | 0/10 | 0/10 | 0 | 0 | Simple | NP | NP | NP | NP | NP |  |
| Thumb abductor muscle (right) | Normal | 0/10 | 0/10 | 0 | 0 | Unable to shrink | NP | NP | NP | NP | NP |  |
| Rectus femoris (right) | Normal | 0/10 | 0/10 | 0 | 0 | Unable to shrink | Normal | 0/10 | 0/10 | 0 | 0 | Unable to shrink |
| Medial head of gastrocnemius muscle (right) | Normal | 0/10 | 0/10 | 0 | 0 | Unable to shrink | NP | NP | NP | NP | NP |  |
| Tibialis anterior muscle (right) | NP | NP | NP | NP | NP |  | Normal | 0/10 | 0/10 | 0 | 0 | Unable to shrink |
| Tibialis anterior muscle (left) | Normal | 0/10 | 0/10 | 0 | 0 | Unable to shrink | Normal | 0/10 | 0/10 | 0 | 0 | Simple |

NP: not performed.

S3-4 Electromyography action unit

| Individuals | II-2 | | | II-3 | | |
| --- | --- | --- | --- | --- | --- | --- |
| Sex/age | Female/29 | | | Male/21 | | |
|  | Average amplitude (uV) | Average time (ms) | Multiphase wave ratio （%） | Average amplitude (uV) | Average time (ms) | Multiphase wave ratio （%） |
| Reference Value | 317.9 | 11.3 | 10.8 | 298.4 | 10.8 | 10.8 |
| Deltoid muscle (left) | 2706 | 18.2 | 40 | NP | NP | NP |
| Deltoid muscle (right) | 5058 | 16 | 0 | 3416 | 15.7 | 0 |
| Reference Value | 308.3 | 11.4 | 7.6 | 293.8 | 11.2 | 7.6 |
| Biceps brachii (left) | 2889 | 15.5 | 0 | NP | NP | NP |
| Reference Value | 264 | 8.9 | 6.4 | 264 | 8.9 | 6.4 |
| Sternocleidomastoid (left) | 743 | 8.7 | 0 | NP | NP | NP |
| Sternocleidomastoid (right) | NP | NP | NP | 697 | 9.2 | 0 |
| Reference Value | 860 | 11.7 | 8.9 | 860 | 11.7 | 8.9 |
| Paraspinal muscle T7 (right) | 4609 | 19.6 | 16.7 | 830 | 15.2 | 30 |
| Reference Value | 725 | 14 | 11.7 | 725 | 14 | 11.7 |
| Iliopsoas muscle (left) | 2356 | 17.3 | 0 | NP | NP | NP |
| Iliopsoas muscle (right) | 711 | 18.2 | 0 | 2570 | 17.9 | 50 |
| Reference Value | 349.9 | 12.0 | 7.3 | 322.4 | 11.3 | 7.3 |
| Rectus femoris (left) | NP | NP | NP | 3130 | 20.1 | 0 |
| Reference Value | 381.3 | 12.7 | 13.9 | 355 | 12.3 | 13.9 |
| Tibialis anterior (left) | NP | NP | NP | 3149 | 17.4 | 87.5 |

NP: not performed.
